# Waterborne Disease Outbreaks Associated With Environmental and Undetermined Exposures to Water — United States, 2013–2014

**DOI:** 10.15585/mmwr.mm6644a4

**Published:** 2017-11-10

**Authors:** R. Paul McClung, David M. Roth, Marissa Vigar, Virginia A. Roberts, Amy M. Kahler, Laura A. Cooley, Elizabeth D. Hilborn, Timothy J. Wade, Kathleen E. Fullerton, Jonathan S. Yoder, Vincent R. Hill

**Affiliations:** ^1^Epidemic Intelligence Service, CDC; ^2^Division of Foodborne, Waterborne, and Environmental Diseases, National Center for Emerging and Zoonotic Infectious Diseases, CDC; ^3^Division of Bacterial Diseases, National Center for Immunization and Respiratory Diseases, CDC; ^4^U.S. Environmental Protection Agency, Washington, D.C.

Waterborne disease outbreaks in the United States are associated with a wide variety of water exposures and are reported annually to CDC on a voluntary basis by state and territorial health departments through the National Outbreak Reporting System (NORS). A majority of outbreaks arise from exposure to drinking water (*1*) or recreational water (*2*), whereas others are caused by an environmental exposure to water or an undetermined exposure to water. During 2013–2014, 15 outbreaks associated with an environmental exposure to water and 12 outbreaks with an undetermined exposure to water were reported, resulting in at least 289 cases of illness, 108 hospitalizations, and 17 deaths. *Legionella* was responsible for 63% of the outbreaks, 94% of hospitalizations, and all deaths. Outbreaks were also caused by *Cryptosporidium*, *Pseudomonas*, and *Giardia*, including six outbreaks of giardiasis caused by ingestion of water from a river, stream, or spring. Water management programs can effectively prevent outbreaks caused by environmental exposure to water from human-made water systems, while proper point-of-use treatment of water can prevent outbreaks caused by ingestion of water from natural water systems. 

CDC analyzed data from waterborne disease outbreaks reported to NORS associated with environmental and undetermined exposures to water during 2013–2014. Outbreaks with an environmental exposure to water are not associated with a recreational water venue or drinking water system, but rather, are linked to other water types including water from cooling towers, industrial processes, agricultural processes, occupational settings, decorative or display settings (e.g., decorative fountains), and water consumed from natural sources such as backcountry streams (*3*). Outbreaks involving an undetermined exposure to water could not be definitively linked to a single type of water exposure because of association with multiple suspected or confirmed water types (e.g., both spa and drinking water systems) or because insufficient epidemiologic, laboratory, or environmental evidence was available to identify the exposure. All outbreaks with first illness onset during 2013–2014 reported by December 31, 2015 are included in this report. NORS defines a waterborne disease outbreak as the occurrence of a similar illness in two or more persons who are linked by time and location to a common water exposure. For each outbreak, data were collected regarding the number of ill persons, hospitalizations, and deaths, along with the sex, age group, symptoms, and duration of illness for persons affected by the outbreak. Results of epidemiologic and laboratory investigations are also reported, including the suspected or confirmed etiologic agent, the type of water to which patients were exposed, and the setting of the water exposure. During the analysis, predominant illness type was assigned, and water type was further categorized as a human-made or natural water system. Human-made water systems include infrastructure intended for water storage or recirculation, whereas natural water systems include raw water that might or might not be treated at the point of exposure. Waterborne disease outbreaks associated with environmental and undetermined exposures to water from prior years have been reported previously (https://www.cdc.gov/healthywater/surveillance/environmental/environ-water-surveillance-reports.html).

## Environmental Exposure to Water

Fifteen outbreaks associated with environmental exposures to water were reported from 10 states during the reporting period (Table 1). A total of 226 cases were identified in association with these outbreaks, with 69 hospitalizations and nine deaths reported. An etiologic agent was confirmed in 14 of 15 outbreaks. *Giardia duodenalis* was determined to be the etiology of seven outbreaks, and *Legionella pneumophila* was implicated in six. No hospitalizations or deaths were reported in association with outbreaks of giardiasis, whereas legionellosis outbreaks accounted for 90% (62 of 69) of hospitalizations and all nine deaths. The remaining seven (10%) hospitalizations were associated with an outbreak involving skin infections caused by *Pseudomonas aeruginosa*. The majority of the reported outbreaks involved either acute respiratory illness (six of 15, caused by *Legionella*) or acute gastrointestinal illness (eight of 15 [seven *Giardia*, one unknown]). *Giardia* was the etiology of six of seven outbreaks linked to a natural water system, whereas *Legionella* caused six of eight outbreaks linked to a human-made water system (Table 2).

## Undetermined Exposure to Water

Twelve outbreaks associated with an undetermined exposure to water were reported from eight states (Table 1), involving 63 cases, 39 hospitalizations and eight deaths. Outbreaks of acute respiratory illness caused by *Legionella* accounted for 11 (92%) of these outbreaks, along with all reported hospitalizations and deaths. One outbreak of acute gastrointestinal illness caused by *Cryptosporidium* was also reported. In addition, one outbreak caused by *Legionella* with first illness onset in 2009 was reported during this reporting period, resulting in nine reported cases and six hospitalizations. Data from this outbreak are presented (Table 1) but not included in the analysis and discussion of outbreaks for 2013–2014.

## Discussion

This summary of waterborne disease outbreaks associated with environmental or undetermined exposure to water features a range of etiologic agents and illustrates that human illness can result from interaction with contaminated water in numerous settings. 

Similar numbers of waterborne disease outbreaks were reported in association with environmental exposures to water linked with human-made (eight outbreaks) and with natural water systems (seven). Six of the outbreaks associated with environmental exposure to water from human-made water systems were caused by *Legionella*. Water management programs can effectively control the growth and spread of *Legionella* in these water systems and are an important tool in outbreak prevention (*6*,*7*). All but one of the outbreaks associated with natural water systems were caused by *Giardia* and involved ingestion of water from a river, stream, or spring. To prevent illnesses and outbreaks in backcountry settings, it is important to appropriately treat water obtained from the natural environment before consuming it. (https://www.cdc.gov/healthywater/drinking/travel/backcountry_water_treatment.html).

Consistent with what has been reported previously (*5*), (11 of 12) outbreaks associated with an undetermined exposure to water in this report were caused by *Legionella*. These outbreaks could not be definitively linked to a single water exposure because they were associated with multiple suspected or confirmed water types (e.g., both spa and drinking water systems were implicated) or because insufficient epidemiologic, laboratory, or environmental data were available to identify a single exposure. Investigations of legionellosis outbreaks exemplify the challenges of determining a single water exposure associated with illness, as *Legionella* can colonize environmental, recreational, and drinking water systems, creating multiple opportunities for susceptible persons to be exposed to contaminated aerosols. All of the legionellosis outbreaks in this report were associated with human-made water systems. Although water management programs are broadly effective for reducing *Legionella* in a majority of these systems, identification of a specific exposure can help identify system control deficiencies and inform timely and targeted remediation to prevent future illness. Furthermore, these challenges underscore the importance of strong partnerships among epidemiology, laboratory, and environmental health practitioners in support of these complex investigations. In this report, *Legionella* was responsible for all nine outbreaks linked to institutional settings and was responsible for all 17 reported deaths and 94% of all reported hospitalizations. This corresponds with surveillance data from drinking water–associated outbreaks (*8*) and underscores the importance of water management programs for maintaining water quality, preventing illness, and saving lives.

The findings in this report are subject to at least one limitation. State and local health departments have varying capacities to detect, investigate, or report these outbreaks. As a result, these data likely underestimate the actual incidence of outbreaks and do not provide an appropriate estimate of the total number of cases or outbreaks for a specific time or location. Despite this limitation, more states reported outbreaks associated with environmental or undetermined exposures to water in this period than did in previous years (27 versus 18 reported for 2011 to 2012 [*1*]).

Outbreak surveillance data play a critical role in waterborne disease prevention for a broad range of pathogens across numerous water types. *Legionella* continues to challenge public health efforts to investigate and prevent outbreaks in human-made water systems and remains a key target for decreasing waterborne disease morbidity and mortality. *Giardia* remains a health risk for persons consuming water from natural water systems and should continue to be an area of focus for health education and prevention activities in these settings. Strong partnerships among epidemiology, laboratory, and environmental health practitioners at all levels are essential for effectively investigating and preventing waterborne disease outbreaks and protecting public health.

**Summary**

**What is already known about this topic?**

Despite ongoing prevention measures, waterborne disease outbreaks caused by environmental exposure to water (linked to water not associated with a recreational water venue or drinking water system) continue to occur. For certain waterborne disease outbreaks, the specific water exposure cannot be determined based on available evidence, including certain *Legionella* outbreaks involving multiple water exposures. CDC collects data on all waterborne disease outbreaks from states and territories through the National Outbreak Reporting System.

**What is added by this report?**

Fifteen outbreaks associated with an environmental exposure to water and 12 outbreaks with an undetermined exposure to water from 2013 to 2014 were reported to CDC, resulting in at least 289 cases of illness, 108 hospitalizations, and 17 deaths. *Legionella* was responsible for 63% of outbreaks, 94% of hospitalizations, and all deaths. All outbreaks of legionellosis were associated with human-made water systems, including infrastructure intended for water storage or recirculation.

**What are the implications for public health practice?**

Waterborne disease outbreaks can be caused by exposure to water in numerous settings. Public health surveillance is important for understanding the incidence of outbreaks associated with environmental and undetermined exposures to water and for prevention of future outbreaks. Based on the outbreaks included in this summary, future prevention measures should focus on water management programs in human-made water systems to control *Legionella *and appropriate point-of-use treatment of raw water from natural water systems before consumption to inactivate *Giardia.*

**TABLE 1:** Waterborne disease outbreaks associated with environmental and undetermined exposures to water* (n = 28), by state or jurisdiction and month of first case onset — Waterborne Disease and Outbreak Surveillance System, United States, 2013–2014

Exposure state/Jurisdiction/Type of exposure	Month	Year	Etiology^†^	Predominant illness^§^	No. cases	No. hospitalizations^¶^	No. deaths**	Water type	Exposure setting
**Environmental**									
Colorado	Oct	2014	*G. duodenalis*	AGI	9	0	0	River/Stream	Park
Illinois	Jan	2013	*P. aeruginosa*	Skin	30	7	0	Other^††^	Store/Shop
Illinois	Sep	2013	*G. duodenalis*	AGI	69	0	0	River/Stream	Park
Michigan	Oct	2014	*G. duodenalis*	AGI	6	0	0	Sewage	Private residence
Minnesota	Dec	2013	*L. pneumophila* serogroup 1	ARI	2	2	0	Ornamental fountain	Casino
Minnesota	Jul	2014	*G. duodenalis*	AGI	6	0	0	River/Stream	Public outdoor area
New Mexico	Jul	2013	*G. duodenalis*	AGI	3	0	0	River/Stream	Camp/Cabin setting
New York	Oct	2013	*G. duodenalis*	AGI	5	0	0	Spring	Other^§§^
Ohio	Jun	2013	*L. pneumophila* serogroup 1	ARI	39	32	6	Cooling tower	Hospital/Health care
Ohio	Jul	2013	*L. pneumophila* serogroup 1	ARI	3	2	1	Cooling tower	Factory/Industrial facility
Ohio	Aug	2014	*L. pneumophila* serogroup 1	ARI	22	8	0	Evaporative condenser/Air conditioner	Church/Place of worship
Pennsylvania	Jul	2013	*L. pneumophila* serogroup 1	ARI	6	3	0	Cooling tower	Prison/Jail (Juvenile/Adult)
Pennsylvania	Aug	2013	*L. pneumophila* serogroup 1	ARI	15	15	2	Cooling tower/Ornamental fountain	Hospital/Health care
Utah	Oct	2014	*G. duodenalis*	AGI	4	0	0	River/Stream	Backcountry
Virginia	May	2013	Unknown	AGI	7	0	0	Lake/Reservoir/Impoundment	Public outdoor area
**Undetermined**									
Alabama	Sep	2013	*L. pneumophila* serogroup 1	ARI	19	14	5	Unknown	Long-term care facility
California	Jan	2014	*L. pneumophila* serogroup 1	ARI	2	2	0	Unknown	Hotel/Motel/Lodge/Inn
California	Apr	2014	*L. pneumophila* serogroup 2–14	ARI	2	2	1	Unknown	Assisted living facility
Kentucky	Jun	2014	*L. pneumophila* serogroup 1	ARI	6	2	0	Unknown	Long-term care facility
Montana	Jul	2014	*Cryptosporidium* sp.	AGI	11	0	0	Unknown	NR
New York	May	2014	*L. pneumophila* serogroup 1	ARI	2	2	0	Unknown	NR
Ohio	Jul	2009^¶¶^	*L. pneumophila* serogroup 1	ARI	9	6	0	Unknown	Long-term care facility
Ohio	Jul	2013	*L. pneumophila* serogroup 1	ARI	2	2	0	Unknown	Indoor Workplace/ Office
Ohio	Mar	2014	*L. pneumophila* serogroup 1	ARI	4	2	1	Unknown	Long-term care facility
Ohio	Apr	2014	*L. pneumophila* serogroup 1	ARI	4	2	1	Unknown	Long-term care facility
Ohio	Oct	2014	*L. pneumophila* serogroup 1	ARI	2	2	0	Unknown	Hospital/Health care
Pennsylvania	Jan	2014	*L. pneumophila* serogroup 1	ARI	6	6	0	Unknown	Assisted living facility
Texas	Apr	2013	*L. pneumophila* serogroup 1	ARI	3	3	0	Unknown	Prison/Jail (Juvenile/Adult)

**Abbreviations:** AGI = acute gastrointestinal illness; ARI = acute respiratory illness; *G. duodenalis* = *Giardia duodenalis*; *L. pneumophila* = *Legionella pneumophila*; *P. aeruginosa* = *Pseudomonas aeruginosa*; Skin = illnesses, conditions, or symptoms related to the skin; NR = not reported.

* The environmental exposure to water category includes outbreaks not associated with exposure to drinking water systems (i.e., public, private or bottled water) or recreational water venues (e.g., swimming pools, lakes). The undetermined exposure to water category includes outbreaks where a single water exposure (i.e., treated or untreated recreational water, drinking water, or environmental exposure) could not be determined based on available evidence.

^†^ Etiologies listed are confirmed, unless indicated “suspected”; for multiple-etiology outbreaks, etiologies are listed in alphabetical order.

^§^ The category of illness reported by ≥50% of ill respondents; all legionellosis outbreaks were categorized as acute respiratory illness.

^¶^ Value was set to “missing” in reports where zero hospitalizations were reported and the number of persons for whom information was available was also zero.

**Value was set to “missing” in reports where zero deaths were reported and the number of persons for whom information was available was also zero.

^††^ This outbreak was associated with a water storage container used in a tattoo and piercing shop.

^§§^ This outbreak was associated with a spring on a private property in a rural area.

^¶¶^This outbreak from 2009 was not included in previous National Outbreak Reporting System reports. Data from this outbreak are presented in this table but not included in the analysis and discussion.

**TABLE 2:** Summary of waterborne disease outbreaks associated with environmental exposures to water and undetermined exposures to water, by *Legionella *and other etiologies — Waterborne Disease and Outbreak Surveillance System, United States, 2013–2014

Outbreak characteristic	Environmental exposures			Undetermined exposures		
	*Legionella,* No. (% of total)*	Other etiology^†^, No. (% of total)*	Total	*Legionella,* No. (% of total)*	Other etiology^†^, No. (% of total)*	Total
**Outbreaks**	6 (40)	9 (60)	**15 **	11 (92)	1 (8)	**12 **
**Cases**	87 (38)	139 (62)	**226 **	52 (83)	11 (17)	**63 **
**Hospitalizations**	62 (90)	7 (10)	**69 **	39 (100)	0 (0)	**39 **
**Deaths**	9 (100)	0 (0)	**9 **	8 (100)	0 (0)	**8 **
**Institutional settings^§^**	3 (100)	0 (0)	**3**	6 (100)	0 (0)	**6**
**Water type**						
Human-made system**^¶^**	6 (75)	2 (25)	**8 **	10 (100)	0 (0)	**10 **
Natural system**	0 (0)	7 (100)	**7 **	0 (0)	0 (0)	**0 **
Unknown**^††^**	0 (0)	0 (0)	**0 **	1 (50)	1 (50)	**2 **

*Percentages are calculated separately for outbreaks associated with environmental exposures to water and outbreaks associated with undetermined exposures to water. These percentages might not sum to 100 because of rounding.

**^†^**Other etiologies include *Giardia*, *Pseudomonas*, *Cryptosporidium*, and unknown.

**^§^**Institutional settings include: hospital/healthcare, long-term care facility, prison/jail.

**^¶^**Includes all outbreaks involving water associated with human-made structures (e.g. infrastructure for water storage or recirculation).

**Includes all outbreaks associated with nonrecreational exposure to water from the natural environment.

**^††^**Human-made versus natural water system is unknown for two outbreaks where the setting was unreported and the water type was undetermined.
